# Dynamical Control of Broadband Coherent Absorption in ENZ Films

**DOI:** 10.3390/mi11010110

**Published:** 2020-01-20

**Authors:** Vincenzo Bruno, Stefano Vezzoli, Clayton DeVault, Thomas Roger, Marcello Ferrera, Alexandra Boltasseva, Vladimir M. Shalaev, Daniele Faccio

**Affiliations:** 1 School of Physics and Astronomy, University of Glasgow, Glasgow G12 8QQ, UK; 2 The Blackett Laboratory, Department of Physics, Imperial College London, London SW7 2BW, UK; s.vezzoli@imperial.ac.uk; 3 Purdue Quantum Science and Engineering Institute, Purdue University, 1205 West State Street, West Lafayette, IN 47907, USA; devaultx@gmail.com (C.D.); aeb@purdue.edu (A.B.); shalaev@purdue.edu (V.M.S.); 4 School of Electrical and Computer Engineering and Birck Nanotechnology Center, Purdue University, 1205 West State Street, West Lafayette, IN 47907, USA; 5 Institute of Photonics and Quantum Sciences, Heriot-Watt University, Edinburgh EH14 4AS, UK; thomas.roger@gmail.com (T.R.); M.Ferrera@hw.ac.uk (M.F.)

**Keywords:** transparent conductive oxide, coherent perfect absorption, epsilon-near-zero media, light-with-light modulation, refractive index change

## Abstract

Interferometric effects between two counter-propagating beams incident on an optical system can lead to a coherent modulation of the absorption of the total electromagnetic radiation with 100% efficiency even in deeply subwavelength structures. Coherent perfect absorption (CPA) rises from a resonant solution of the scattering matrix and often requires engineered optical properties. For instance, thin film CPA benefits from complex nanostructures with suitable resonance, albeit at a loss of operational bandwidth. In this work, we theoretically and experimentally demonstrate a broadband CPA based on light-with-light modulation in epsilon-near-zero (ENZ) subwavelength films. We show that unpatterned ENZ films with different thicknesses exhibit broadband CPA with a near-unity maximum value located at the ENZ wavelength. By using Kerr optical nonlinearities, we dynamically tune the visibility and peak wavelength of the total energy modulation. Our results based on homogeneous thick ENZ media open a route towards on-chip devices that require efficient light absorption and dynamical tunability.

## 1. Introduction

Coherent perfect absorption (CPA) was first proposed as a time-reversed version of a laser [1]. Similar to a laser cavity, CPA occurs when light is resonant at specific wavelengths in a high-Q Fabry–Perot optical resonator. However, for CPA, the active gain material is replaced with a moderately lossy medium. Because the system’s single-pass losses are typically low, perfect absorption for a given input intensity is extremely sensitive to the Q-factor and resonance wavelength [2,3].

An alternative scheme utilizes deeply subwavelength and highly absorbing materials [4,5,6]. Here, two counter-propagating coherent beams interfere at the film’s surface and create a standing wave. Absorption in the film is then modulated by changing the relative phase of the two beams, or equivalently by scanning the film along the nodes (peak transmittance) and antinodes (peak absorption) of the interference pattern. This approach has been demonstrated in the ultrafast [7] and quantum regime [8,9,10,11], as well as in integrated photonic systems [12,13,14,15,16]. While a resonant cavity is not required, single-pass absorption should be 50% to achieve perfect absorption [17,18]. This is difficult to obtain in conventional dielectrics (too little losses) or metals (too high reflectivity). To circumnavigate this challenge, metasurfaces—nanostructured subwavelength films—with ideal absorptive optical properties have been used to achieve CPA [4,19]. Ideal absorption can be achieved in extremely subwavelength films over a broad range of wavelengths, making metasurface-based CPA advantageous over bulk cavity structures and compatible with integrated photonic platforms [3].

While metasurfaces and other engineered structures can exhibit CPA over large wavelength ranges, the necessary nanofabrication can be a limitation for practical CPA applications. Thin films of epsilon-near-zero (ENZ) materials, such as transparent conductive oxides (TCOs) like aluminum-doped zinc oxide (AZO) or indium tin oxide (ITO), have been proposed as a particularly suitable platform for broadband CPA [20]. ENZ materials exhibit a real part of the dielectric permittivity which crosses zero for wavelengths of practical interest in the near-infrared or visible regions [21,22]. Due to the continuity of the transverse component of the electric field at the interface, the electric field within the ENZ material can be very large and can lead to perfect absorption (PA) when illuminated at a critical angle of incidence [23,24]. In the limit of deeply subwavelength ENZ film, PA is provided by critical coupling the incident light to a fast wave propagating along the ENZ layer [24]. The proposed systems for ENZ PA are multilayer structures where the ENZ thin layer is sandwiched between two dielectrics or a dielectric and a metal structure [25]. At the critical angle where CPA happens (this is often referred to as directional PA), the loss follows a linear relationship with the ENZ film thickness which implies that CPA can occur in an arbitrarily thin ENZ film (with arbitrary small single-pass absorption) [26]. For instance, PA has been demonstrated for films of ITO film thickness as low as 0.02 $\lambda_{0}$ (free-space wavelength) and with only 5% single-pass absorption [27]. Electrical tuning of one port directional PA have also been shown in plasmonic strip cavity based on a ENZ thin layer, with a modulation in reflectance of the 15% [28]. Finally, broadband coherent modulation of directional PA in ENZ deeply subwavelength film have been proved by using ITO multylayer structures sandwiched between two ZnSe prisms [20]. The control of nonlinear processes by two port illumination was also theorized for deeply sub-wavelength ENZ slab [29]. Applications of CPA in deeply subwavelength ENZ films could be found in photovoltaic energy conversion or devices such as bolometers which require large absorption with small masses. However, other applications, such as in nonlinear or quantum optics, may benefit from thicker films where the efficiency of the nonlinear process and the parametric gain generally scale with thickness.

Here, we study CPA in films of TCOs near their ENZ wavelength where the film’s refractive index exhibits large anomalous dispersion and a near-zero refractive index. Such films can be treated as deeply subwavelength because the effective wavelength will increase drastically for wavelength approaching the ENZ wavelength. We theoretically and experimentally explore the role of this transition region in order to achieve CPA in homogeneous AZO optically thick films and then show how this can be controlled with intense optical pump fields. It was recently shown that the combination of low refractive index and the high damage threshold of these materials allows TCOs to exhibit large and ultrafast Kerr-type optical nonlinearities in the ENZ region [30,31,32,33,34,35,36] and behave as efficient time-varying medium [37,38].

We perform CPA experiments in a Sagnac-like interferometer where two counter propagating light pulses are incident normal to the sample. We achieve coherent control of absorption in AZO films with different thicknesses. For all samples the total energy modulation exhibits a maximum value near the ENZ wavelength. We then demonstrate dynamical control of CPA using its strong intensity-dependent refractive index change. Our demonstration of broadband and tunable CPA in homogeneous ENZ films is relevant for practical nanoscale optical-switches and modulators where alternative nano-pattered metasurfaces would suffer from low switching efficiencies and detriments of nanofabrication processes.

## 2. Theoretical Investigation

**Figure 1:**
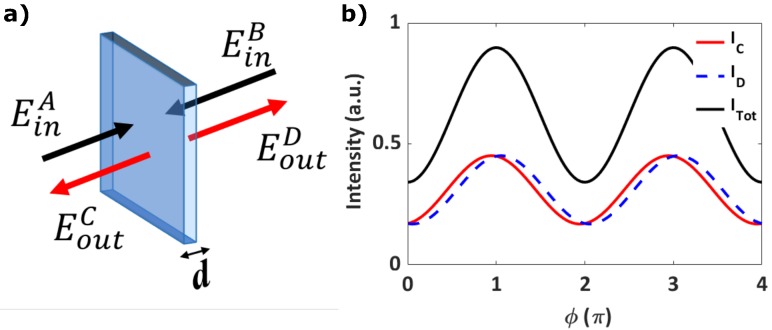
(**a**) Bi-directional coherent perfect absorption (CPA) scheme. (**b**) Intensity of the two output beams, C and D, and its sum as we scan the sample position in the propagation direction. This is equivalent to changing the relative phase between the two input fields ϕ.

Our optical system consists in two counter-propagating continuous waves (CW) impinging on a homogeneous ENZ film, $E_{in}^{A}$ and $E_{in}^{B}$, respectively, at normal incidence (Figure 1a). From the transfer matrix method (TMM), we calculate the electric field at the two outputs of our symmetric system, $E_{out}^{C}$ and $E_{out}^{D}$ respectively. By changing the relative phase ϕ between the two counter-propagating beams, we simulate the scenario in which the film is shifted along the propagation direction and calculate the intensity of the two outputs, C and D (Figure 1b). By summing the intensity at the two outputs ($I_{Tot}=I_{C}+I_{D}$), we define the modulation visibility of the total energy as

(1) $$V_{tot}=\frac{\left(I_{Tot}^{max}-I_{Tot}^{min}\right)}{\left(I_{Tot}^{max}+I_{Tot}^{min}\right)}$$

where $I_{Tot}^{max}$ and $I_{Tot}^{min}$ are the maximum and minimum of the total output energy of the system. In principle, for CPA to occur in a thin film, the transmission and reflection coefficients from both sides of the film should be equal (|r|=|t|) with a phase difference of $\varphi_{rt}=0$ or π in order to achieve 100% light absorption. In this situation, the value of the total visibility is 1.

**Figure 2:**
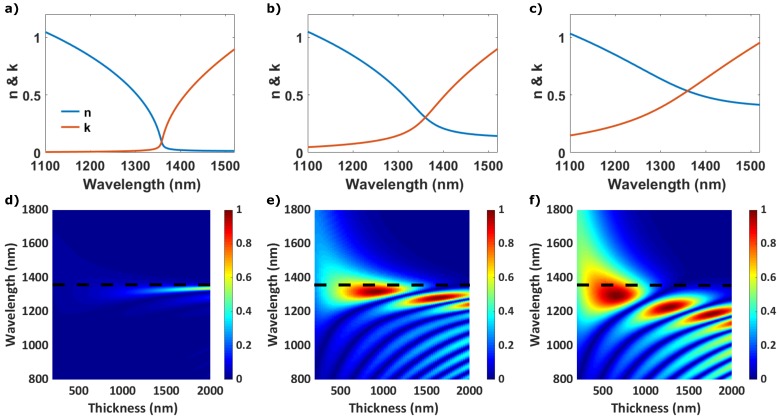
(**a**–**c**) Real and imaginary part of the refractive index of the three cases with $\lambda_{ENZ}$ ≈ 1350 nm. (**d**–**f**) Normalized visibility of the total energy as a function of the wavelength for different thicknesses. The dashed red line indicates the $\lambda_{ENZ}$. For the dispersion we use $\epsilon_{\infty }=3.18$ and $\omega_{p}$ = 2.4745 × 10^15^ rad/s. For the damping constant we use $\gamma_{a}=1.0073\times 10^{13}$, $\gamma_{b}=0.8053\times 10^{14}$ and $\gamma_{c}=2.3614\times 10^{14}$ rad/s.

We consider three different cases by fixing the zero crossing of the real part of the dielectric permittivity at $\lambda_{ENZ}\approx$ 1350 nm, but vary the dispersion across the ENZ region as shown in Figure 2a–c where we plot the refractive index profiles (real, *n*, and imaginary, *k*, parts) for three different cases studied. These are calculated from a Drude model

(2) $$\epsilon =\epsilon_{\infty }-\frac{\omega_{p}^{2}}{\left(\omega^{2}+i\gamma \omega \right)}$$

where $\epsilon_{\infty }$ is the high frequency permittivity, $\omega_{p}$ is the plasma frequency and γ is the damping coefficient. It has been shown that this model correctly reproduces the ENZ refractive index for a variety of materials, as ITO and AZO [33,39,40,41]. In our case, we use $\epsilon_{\infty }=3.18$ and $\omega_{p}$ = 2.4745 × 10^15^ rad/s. We vary the Drude model damping coefficient, thus increasing losses and reducing the dispersion gradient in *n*, from (a) to (c) γ = 1.0073 × 10^13^ → 2.4745 × 10^14^ rad/s. Figure 2d–f show the visibility $V_{tot}$ of the total energy as a function of the ENZ film’s thickness for the three cases shown in Figure 2a–c. In Figure 2d ($\gamma_{a}$), we do not observe coherent modulation of the total energy for thickness below 1000 nm. Due to the high transmission of the thin film, the interference between the reflected and transmitted field is weak. For thicker films, *r* and *t* become more similar and stronger interference is observed. The TMM model predicts visibility with a maximum value close to one that is pinned to a wavelength slightly shorter than $\lambda_{ENZ}$. When we increase the optical losses of the ENZ slab, Figure 2e,f, the peak of the visibility becomes broader, exhibiting multiple resonances as the thickness increases but all with maximum absorption at a wavelength just below $\lambda_{ENZ}$. These results show that the system exhibits broadband coherent modulation of the energy with a maximum value close to one just below $\lambda_{ENZ}$, independently of the thickness and of the single-pass absorption. We associate this maximum to a Fabry–Perot (FP) like resonance due to interference effects in the Air/AZO/glass system. The fact that the FP resonance is ‘locked’ before the ENZ wavelength irrespectively of the thickness is due to the ENZ condition [42,43].

In the ideal case without losses, the first resonance of an FP cavity is reached when the $2nd=\lambda_{0}$. Due to the strong gradient of the *n* before the ENZ region, the $\lambda_{0}$ at which the first resonance occurs will not scale linearly with *d*, but it will be locked in this spectral range with strong dispersion. Moreover, in a lossy dielectric medium, *r* and *t* become complex and their phases depend on the value of both *n* and *k* of the lossy medium. Here the first resonant order for the FP cavity is reached when $2nd=\lambda_{0}\left(1-\alpha /\pi \right)$, where α is the phase of the transmission coefficient [18]. Combining the strong dispersion of *n* due to the ENZ condition and the value of *k*, almost perfect modulation of absorption is expected at wavelength just below $\lambda_{ENZ}$ even for subwavelength thickness (Figure 2f).

## 3. Coherent Absorption and Its Dynamical Control

**Figure 3:**
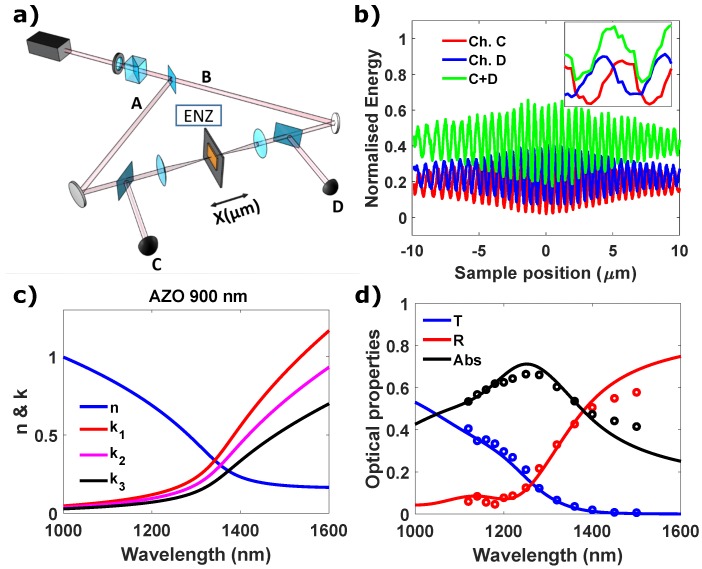
(**a**) Schematics of the Sagnac interferometer. (**b**) An example of measurement for $\lambda_{0}$ = 1280 nm, assuming energy equal to 1 at the interferometer input. The total modulation of the energy (or absorption) is given by the sum of C and D (green curve). The inset shows a zoom of the interferogram. (**c**) ellipsometer measurement of the index of refraction of AZO 900 nm thick film, (**d**) experimental (dots) and TMM simulation (solid line) of R, T and abs for the same sample.

We experimentally investigated the behaviour of CPA in ENZ films using AZO films illuminated by two counter-propagating laser beams in a Sagnac-like interferometer configuration. Figure 3a shows a schematic of the set-up. Laser pulses (105 fs FWHM duration, repetition rate 100 Hz) are generated by an Optical Parametric Amplifier (TOPAS) in a tunable range between 1120 nm and 1500 nm. The input power is controlled through a half wave plate and a polarizing beam splitter, which also fixes the input p-polarization (horizontal in the lab frame). The beam is split by a non-polarizing beam splitter into two beams A and B with equal energy and then recombined onto the sample at normal incidence. The AZO film (deposited on a 1 mm thick glass slide) is facing the beam A, whereas the beam B is incident on the substrate side. The two beams are focused down to 50 µm by using a pair of 125 mm lenses. By moving the sample with a piezo-electric stage, interferograms are generated at the output C and D and measured with photodiodes. We used two beam splitters to extract the light from the interferometer and send it to the photodiodes. A representative example of these interferograms are shown in Figure 3b. In order to calculate the energy visibility in the pulsed case we proceed in the same way as for the CW case, i.e. we evaluate the central portion (where the pulse intensity is maximum) of the interferogram and extrapolate the average values for maximum and minimum of the intensity.

We investigated two AZO samples of similar optical properties, i.e., *n* and *k*, in the ENZ region, but with thicknesses of 500 and 900 nm (Figure 3c). The real part of the dielectric permittivity crosses zero around 1340 nm for the 900-nm-thick film, which corresponds to where the real and imaginary part of the refractive index are equal ($n_{900}=k_{900}=0.34$). For the 500-nm-thick sample the $\lambda_{ENZ}$ is redshifted by 30 nm ($n_{500}=k_{500}=0.52$ at the $\lambda_{ENZ}$) due to small differences in the material deposition. In the spectral range under analysis, *n* of the AZO 900-nm-thick film passes from close to 1 around 1100 nm to less than 0.2 for longer wavelengths. Since $\lambda_{eff}=\lambda_{0}$/*n* inside the medium, the effective length ($L_{eff}=L/\lambda_{eff}$) of our sample is 0.8 $\lambda_{0}$ at 1050 nm, 0.21 $\lambda_{0}$ at the ($\lambda_{ENZ}=1350$ nm, and becomes optically deeply subwavelength around 1500 nm (0.1 $\lambda_{0}$ ).

We also deposited three 900-nm-thick AZO films on a glass substrate three samples with similar *n* (about 20% difference), but different value of *k* at the crossing point ($k_{1}=0.34$, $k_{2}=0.30$ and $k_{3}=0.27$ for the 900 nm thick film). All the samples exhibit similar optical properties with an absorption close to 60% across the ENZ region (Figure 3d).

**Figure 4:**
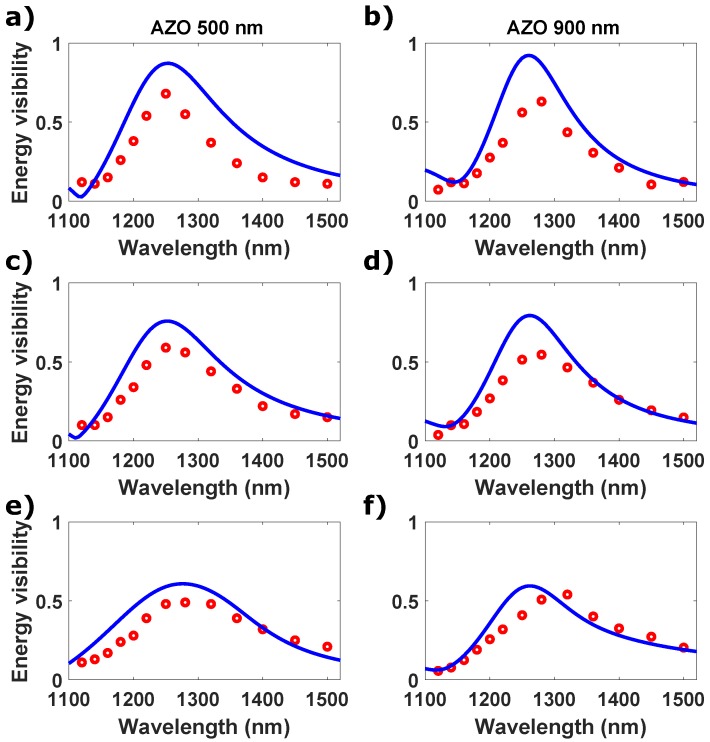
Experimental (circles) and transfer matrix method (TMM) simulation (solid line) of normalized visibility of the total energy for aluminum-doped zinc oxide (AZO) 500 nm and 900 nm with different values of *k*. (**a**,**b**) High losses $k_{1}$, (**c**,**d**) middle losses $k_{2}$ and (**e**,**f**) low losses $k_{3}$. For the TMM simulation we suppose Δλ∼60 nm.

We first perform a CPA experiment for the bare glass substrate. In this case the energy modulation is almost zero for all the spectral range of interest. In Figure 4 we report the measured normalized total energy modulation visibility (red circles), together with the values predicted by the TMM (solid lines) for the AZO film. All the samples show the same trend independently from the thickness. In the case of high optical losses, for the different thicknesses the visibility is almost zero in the region where the index of refraction is close to one, whereas it increases and reaches a maximum value up to the 60% just before $\lambda_{ENZ}$. As we decrease the value of *k*, the trend of the visibility remains the same for all the samples, but its maximum value across the transition region decreases. Overall, the experimental results confirm the predictions that CPA can be observed in ENZ films over a broad bandwidth with thicknesses larger than the conventional subwavelength designs. The bandwidth of ∼100 nm is comparable with CPA in deeply subwavelength ENZ single layer (∼150 nm [20]) or white-light cavity (∼100 nm [3]), whereas it is larger than metasurfaces (∼40 nm [4]).

**Figure 5:**
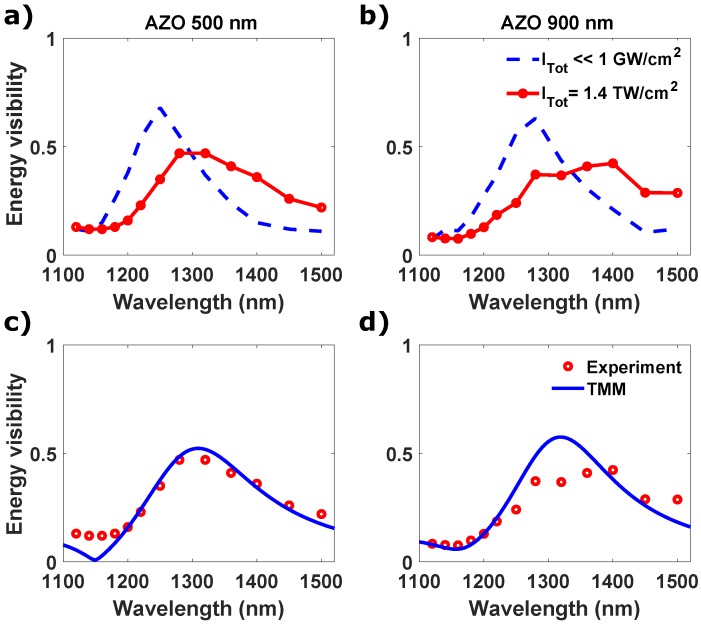
(**a**,**b**) Normalized visibility of the total energy for both samples, 500 nm (**a**) and 900 nm (**b**). The dashed blue curve represents the linear characterization, while the circles is the nonlinear CPA with high beam intensity. (**c**,**d**) Experimental (circles) and TMM simulation (solid line) of normalized visibility of the total energy for AZO 500 nm and 900 nm for the nonlinear CPA.

We finally investigate nonlinear coherent absorption in ENZ films based on modification of the film refractive index through the nonlinear Kerr coefficient. Previously it has been demonstrated that the ENZ condition leads to the enhancement of third order nonlinearities in terms of nonlinear refractive index change for thin film of AZO [32]. This is based on the observation that when the permittivity is close to zero, any nonlinear change Δn, proportional to $\chi^{\left(3\right)}/n$, is enhanced due to the *n* tending to low values. In Ref. [32] a refractive index change of 400% was reported for an AZO film optically pumped with 1.3 TW/cm^2^ without showing damage of the sample or saturation of the optical Kerr effect. In the same work, at λ = 1310 nm a nonlinear susceptibility of $Re\left[\chi^{\left(3\right)}\right]∼4.73\times 10^{-20}$ V^2^/m^2^ and $Im\left[\chi^{\left(3\right)}\right]∼0.57\times 10^{-20}$ V^2^/m^2^ was extrapolated. We therefore illuminated the AZO film in the Sagnac interferometer with two high intensity pulses at normal incidence and same wavelength. The intensities on each side are 0.8 and 0.6 TW/cm^2^, respectively. By increasing the intensities from the linear regime to these maximum values, we observe that the CPA visibility passes from 68% of the linear case to 35% (Figure 5a,b). The peak of the normalized visibility also redshifts and becomes broader for both the samples, with a nearly 50 nm-shift for the 500 nm sample. Following the recent works in TCOs, this can be explained by the fact that the dielectric permittivity, and so the optical constants including $\lambda_{ENZ}$, exhibit a redshift when it is optically pumped across the ENZ wavelength [31,44]. The redshift of the $\lambda_{ENZ}$ is also associated to a positive Δn and to a negative Δk [30,32]. Due to the decreasing of *k*, the visibility drops, as we observed for the linear case. While, the shift of the visibility peak is related to the shift of $\lambda_{ENZ}$ in the same direction, and therefore to the shift of the strong dispersion which the material exhibits at wavelength shorter than the zero-crossing frequency. In Figure 5c,d we plot the experimental results together with TMM simulations. The TMM simulations are obtained considering a ∼60 nm shift of $\omega_{p}$ and a decreasing of γ ($0.15\times 10^{15}$→$0.09\times 10^{15}$). This correspond to a $\Delta \lambda_{ENZ}∼60$ nm and to a $k_{500}=0.38$ and $k_{900}=0.24$. These results show that enhanced nonlinearities in ENZ materials can be used to add a degree of freedom to tune the efficiency and the bandwidth of coherent absorption.

## 4. Conclusions

We theoretically and experimentally demonstrate coherent control of absorption in films of ENZ material. We show that it is possible to achieve a coherent absorption-mediated interferometric effect with a maximum of its effect locked just below the $\lambda_{ENZ}$ wavelength. Due the strong dispersion at wavelengths below the crossing point, it can be tuned to any wavelength shorter than $\lambda_{ENZ}$ by varying film thickness and the optical losses. The 60% total visibility achieved in the AZO film could be improved using a CW and collimated beam in order to achieve CPA. By using AZO’s strong intensity-dependent nonlinearities, we also showed that it is possible to dynamically tune the visibility of the total energy by simply increasing the intensity of the incoming beam. The possibility to add a degree of freedom for the coherent control of the absorption in ENZ media by using intensity-dependent refractive index proposes a route towards technologies [45] such as optical data processing or devices that require efficient light absorption and dynamical tunability. All the data supporting this manuscript are available at http://researchdata.gla.ac.uk/939/.
